# The complete chloroplast genome sequence of *Thalictrum aquilegiifolium* var. *sibiricum* (Ranunculaceae)

**DOI:** 10.1080/23802359.2022.2088309

**Published:** 2022-06-28

**Authors:** Kanae Michimoto, Takuro Ito, Masayuki Maki

**Affiliations:** Botanical Gardens, Tohoku University, Aoba, Sendai, Japan

**Keywords:** Chloroplast, meadow rue, phylogenetic analysis, Ranunculaceae, *Thalictrum*

## Abstract

*Thalictrum aquilegiifolium* (Ranunculaceae) is widely distributed in the Eurasian Continent and Japan and comprises some intraspecific taxa. We report here the complete chloroplast genome of *T. aquilegiifolium* var. *sibiricum*. The plastome of *T. aquilegiifolium* var. *sibiricum* is 156,074 bp in length, containing large (85,457 bp) and small (17,642 bp) single-copy regions which are separated by a pair of inverted repeats (26,487 bp each). The genome consists of 119 genes, including 88 protein-coding, four ribosomal RNA genes, and 27 transfer RNA genes. Our phylogenetic analysis revealed that *Thalictrum* species formed a highly supported clade, indicating that these species are monophyletic.

The genus *Thalictrum* (Ranunculaceae) consists of 120-200 species that are globally distributed (Park and Festerling 1997). This genus is considered an ideal group in which to examine correlated evolution of polyploidy, sexual system, and pollination mode because it includes species with large variations in these traits (Soza et al. 2013). Some species, such as *Thalictrum filamentosum* Maximowicz 1855, also have horticultural value. However, this genus is taxonomically difficult, and its taxonomic treatment requires careful examination using population-based field studies (Park and Festerling 1997).

*Thalictrum aquilegiifolium* Linnaeus 1753 is distributed from Europe to East Asia. Two varieties are found in Japan, var. *sibiricum* Regel et Tiling 1858 and var. *intermedium* Nakai 1880 (Kadota 2016). While *T. aquilegiifolium* var. *sibiricum* is widely distributed in Far East Asia, including China, Korea, and Japan, *T. aquilegiifolium* var. *intermedium* is found only in Japan. The former variety is designated as a threatened species (category IB, Endangered species (EN)) in the national Red Data Book of Japan (Japanese Ministry of the Environment 2015). Because these two varieties are distinguished only by the number of achenes, they may be misclassified when the achenes are immature. Therefore, it is necessary to accurately identify each variety for conservation purposes. Genetic information will be useful for distinguishing the varieties.

Chloroplast (cp) genome sequences are considered useful for molecular phylogenetics (Jansen et al. 2007), DNA barcoding (Hollingsworth et al. 2011), population genetics (Powell et al. 1995), and transplastomic studies (Bock and Khan 2004). Here, we characterize the complete cp genome of *T. aquilegiifolium* var. *sibiricum* based on Illumina paired-end sequencing data. Furthermore, by incorporating the cp genome sequences published to date into a phylogenetic analysis, we reconstruct the phylogeny of Ranunculaceae and examine the phylogenetic position of *T. aquilegiifolium* var. *sibiricum*.

Total genomic DNA was extracted from *T. aquilegiifolium* var. *sibiricum* collected from a population in Marumori-cho Town, Miyagi Prefecture, Japan (37°52′N, 140°45′E) using a modified CTAB method (Doyle and Doyle 1987). A voucher specimen (K. Michimoto-1) is deposited in the herbarium of the Botanical Gardens, Tohoku University (TUS; contact Takuro Ito: takuro.ito.c4@tohoku.ac.jp). The purified genomic DNA was subjected to paired-end 150 bp sequencing using the Illumina HiSeq X platform (Macrogen, Tokyo, Japan). The raw data (707,458 reads) were assembled using NOVOplasty (Dierckxsens et al. 2017) with the cp genome sequence of *Thalictrum minus* Linnaeus 1753 (GenBank accession number: NC_041544) as a reference. The complete cp genome was annotated using Geseq version 2.03 (Tillich et al. 2017). The circular genome map was visualized using OGDRAW version 1.3.1 (Greiner et al. 2019). The annotated plastome sequence was deposited in GenBank (accession number: LC661621).

The complete plastome of *T. aquilegiifolium* var. *sibiricum* is 156,074 bp in length, including two single-copy regions (large single-copy region, LSC: 85,457 bp and small single-copy region, SSC: 17,642 bp) and two inverted repeat regions (IRs: 26,487 bp each). The nucleotide composition is asymmetric (30.48% A, 19.56% C, 18.81% G, and 31.15% T) with an overall G + C content of 38.37%. The complete cp genome of *T. aquilegiifolium* var. *sibiricum* contains 119 genes in total, including 88 protein-coding genes, 27 tRNA genes, and 4 rRNA genes.

To reveal the phylogenetic position of *T. aquilegiifolium* var. *sibiricum*, 25 other complete cp genomes of Ranunculaceae including the *Thalictrum* species examined so far were aligned with *T. aquilegiifolium* var. *sibiricum* using MAFFT (Katoh and Standley 2013) and trimmed using trimAL version 1.2 (Capella-Gutiérrez et al. 2009). A maximum likelihood analysis was performed using raxmlGUI 2.0 (Edler et al. 2021) with 1000 bootstrap replicates. *Glaucidium palmatum* Siebold et Zucc. 1845 was set as an outgroup because this species is often treated as a member of the Hydrastidaceae, which is considered a sister group of the Ranunculaceae (Loconte et al. 1995; Stevens 2001 onwards).

The result showed that *Thalictrum* comprises a monophyletic group with a 100% bootstrap value (Figure 1). Because two *T. aquilegiifolium* in the present and previous study did not form a monophyletic group, it will be necessary to consider past gene flow between the species and others or to reconsider the taxonomic treatments of the two populations. *Leptopyrum* and *Thalictrum* formed a fully supported sub-clade which formed a fully supported clade with another sub-clade including *Semiaquilegia*, *Urophysa*, and *Enemion*, consistent with the conclusion that these genera belong to the subfamily Thalictroideae. The complete plastome sequence of *T. aquilegiifolium* var. *sibiricum* revealed in this study will provide important genetic information for future evolutionary studies of Ranunculaceae.

**Figure 1.**
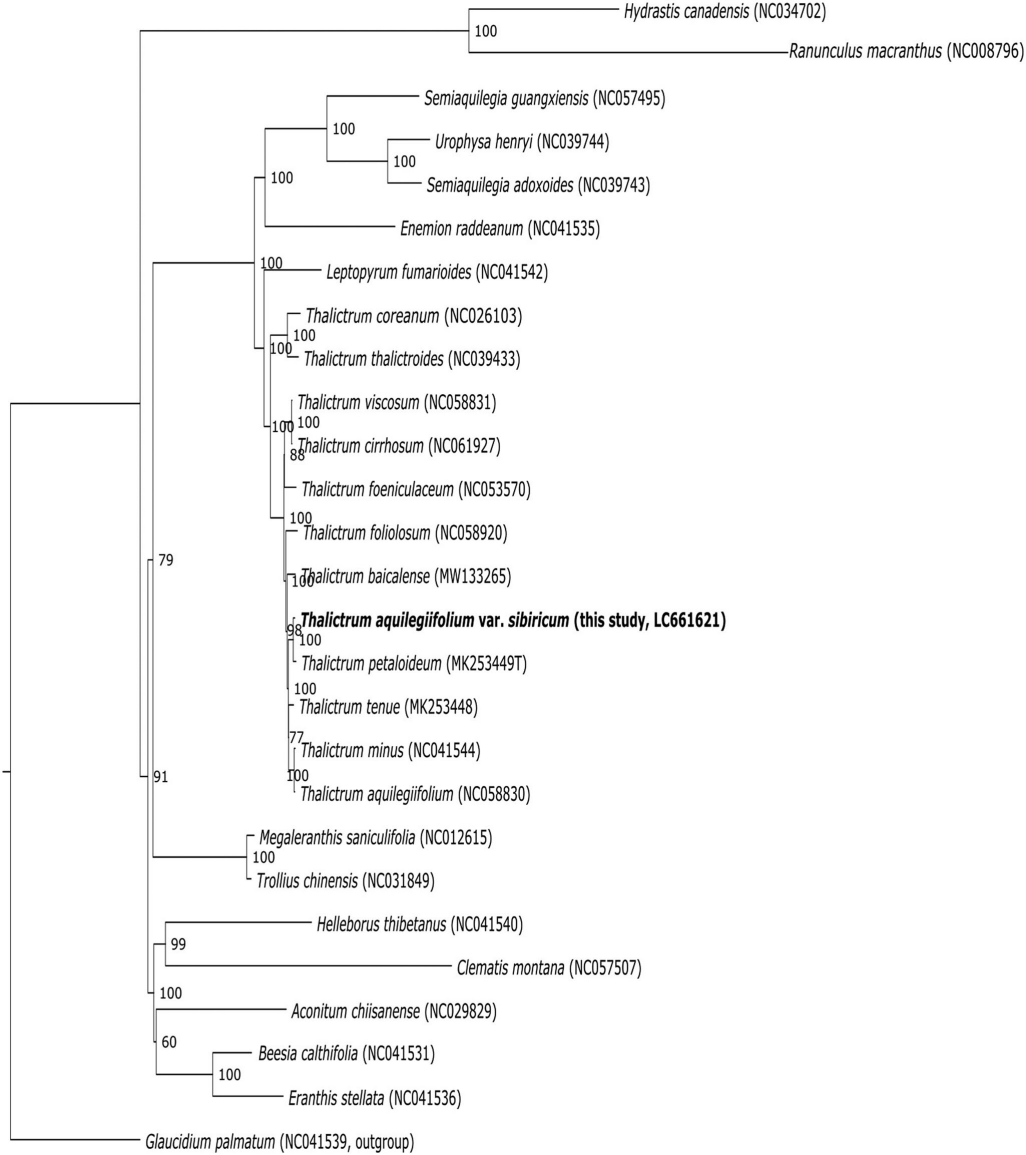
Maximum likelihood tree of *T. aquilegiifolium* var. *sibiricum* and other Ranunculaceae species based on complete chloroplast genome sequences, with *Glaucidium palmatum* as the outgroup. Bootstrap support values (based on 1000 replicates) are shown adjacent to the nodes.

## Data Availability

The genome sequence data that support the findings of this study are openly available in DDBJ at http://getentry.ddbj.nig.ac.jp/top-j.html under accession no. LC661621. The associated BioProject, SRA, and Bio-Sample numbers are PRJDB12595, DRR328409, and SAMD00424020, respectively.
